# A New Perforator Flap From Distal Medial Arm: A Cadaveric Study

**Published:** 2010-10-18

**Authors:** Yakup Cil, Necdet Kocabiyik, Serdar Ozturk, Selcuk Isik, Hasan Ozan

**Affiliations:** ^a^Department of Plastic Surgery, Eskisehir Military Hospital, 26020 Eskisehir, Turkey; ^b^Department of Anatomy, Gulhane Military Medical Academy, 06018 Ankara, Turkey; ^c^Department of Plastic Surgery and Burn Center, Gulhane Military Medical Academy, 06018 Ankara, Turkey

## Abstract

**Background:** In this study, a new fasciocutaneous perforator flap raised from inner medial surface of the distal medial arm has been described for soft tissue coverage. **Methods:** The blood supply of this flap comes directly from the distal brachial artery. Fourteen limbs of 7 formalinized cadavers were dissected to study the origin and the course of perforator vessel. **Results:** The average size of the flap was 10.7 cm × 5.6 cm in the distal medial arm region. The constant main perforator was measured within a circle of 2.76-cm diameter, the center of which was 11.5 cm above and 1.3 cm medial to the medial epicondyle of humerus. The mean length and diameter of the distal brachial perforator artery were 3.3 cm and 0.95 mm, respectively. **Conclusion:** We think that this flap may be a useful option for the reconstruction of soft tissue defect of elbow.

Elbow's contractures and skin defects are commonly seen.^1^ Various methods have been published regarding reconstruction of the skin defects for the elbow region such as skin grafts, local and distant skin flaps,^1^ muscle and musculocutaneous flaps,^2^ and free flaps.^3^ Skin grafting has been the most widely accepted method, but it often produces a patchy appearance and requires prolonged splinting in order to avoid further recurrence. Furthermore, it cannot be applied to most of the complicated contractures. Local flap, especially the fasciocutaneous flap, when available, facilitates closure of the tissue defect for elbow region considerably. Advantage of the fasciocutaneous flap usage has been widely accepted for several conditions.^4^,^5^ The medial distal arm has been described as a potential donor site for the fasciocutaneous flap because of its excellent color, fine texture, and ideal thickness.^6^ We have recognized the distal brachial artery's main perforator (DBAMP) while raising the ulnar recurrent fasciocutaneous island flap (Fig 1). We planned this cadaveric study to demonstrate the characteristics of the distal brachial artery's main perforator fasciocutaneous flap (DBAMPFF).

## MATERIALS & METHODS

**Figure 2:**
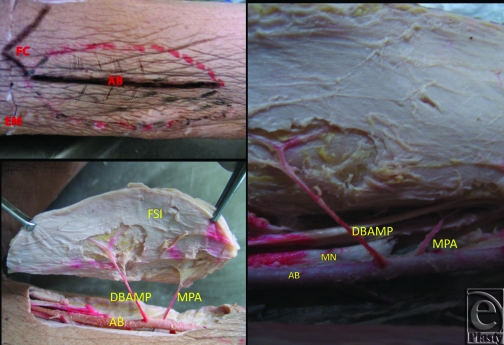
View of DBAMPFF at the right upper limb of cadaver. Main perforator vessel of DBAMPFF and small perforator are also seen. Abbreviations: AB, arteria brachialis; DBAMP, distal brachial artery's main perforator; FSI, flap skin island; MN, median nerve; MPA, minor perforator artery.

In this study, 14 upper limbs of 7 formalinized cadavers were studied. First, midline incision was performed in the axillary fossa to identify the brachial artery. The brachial artery was exposed. After cannulating the artery at the lower margin of the teres major muscle, 20~ml India ink dye was injected into the brachial artery until skin staining became evident. Afterwards, the flap was raised by incising the margins of the stained skin. Initially, surgical loop was used to elevate skin margin of the flap. The incision was carried down through subcutaneous fat. Nervus cutaneous antebrachii medialis was seen the flap undersurface clearly. Nervus cutaneous antebrachii medialis protected and flap harvesting was continued down to the deep fascia of the distal medial arm. The intermuscular septum between the triceps and brachialis was released, carefully. The deep distal incision was made, severing distal brachial artery's distal perforator vessels. Main perforator artery of distal brachial artery was seen on the lateral side of the median nerve. Dissection was continued by use of operating microscope when the perforator artery's origin of the skin branch was identified (Fig 2). The distance between medial epicondyle of humerus and the origin of the main perforator artery from the distal brachial artery was measured with digital caliper (Absolute Digimatic Caliper-Mitutoya Solar, PennTool Co, Maplewood, New Jersey). Distal brachial artery main perforator vessel's diameter (mm), vessel's length (cm), the distance between the epicondylus medialis of humerus and the skin entrance of perforator artery; and flap skin territory were measured.

## RESULTS

**Figure 3:**
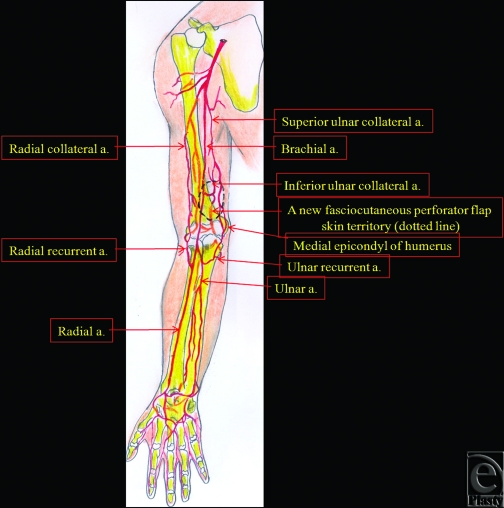
Schematic representation of the distal brachial artery's main perforator fasciocutaneous flap before flap elevation (dotted line).

**Figure 4:**
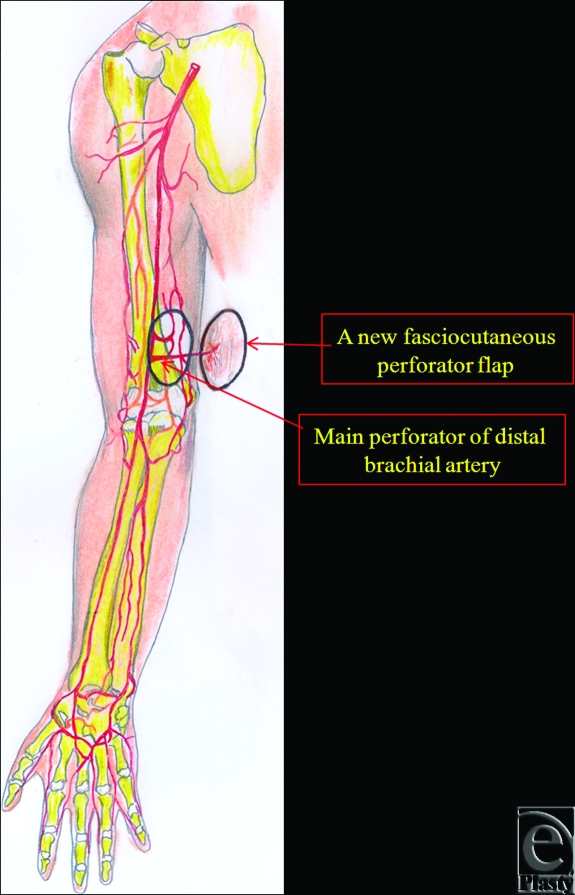
Schematic representation of the distal brachial artery's main perforator fasciocutaneous flap after flap elevation.

All upper arms were dissected by the same surgeon in the laboratories of anatomy department. The distal brachial artery main perforator vessel was seen in all dissected cadavers. In all cadavers, the flaps based on the distal brachial artery main perforator vessels were successfully raised on the medial site of distal arm. Flap dissection was a relatively simple procedure. At least, 1 additional small perforator artery was identified in all cadavers (Fig 2). The main perforator was measured within a circle of 2.76-cm diameter, center of which was 11.5 cm above and 1.3 cm medial to the medial epicondyle of humerus (Figs 3 and 4). The mean length and diameter of the DBAMP were 3.3 cm and 0.95 mm, respectively. The mean skin territory was 10.7 cm × 5.6 cm. The DBAMPFF characteristics are shown in Table 1.

## DISCUSSION

Surgeons have preferred the perforator flaps because of minimal donor site morbidity, color, texture, and flap thickness for many years.^7^^-^9

Elbow contractures can be treated with various methods. When the flexion contracture of elbow is limited with a solitary longitudinal scar band, Z-plasties are often recommended, but they sometimes fail to release the entire contracture; on the other hand, linear band contractures are uncommon at the elbow.^6^ Mostly, replacement of the skin of elbow contracture is necessary for permanent and satisfactory results.

Although, skin grafts can safely restore normal length when the contracted scar tissue has been adequately removed, they are troublesome, because they often produce a patchy appearance and require prolonged splinting in order to avoid further recurrence.^10^ Furthermore, they cannot be applied at all to complicated burn contractures. If vital structures have been exposed, flap repair becomes the unique alternative.

Direct-distant flaps from the ipsilateral chest or flank can be selected.^7^ The disadvantages of using these flaps are the need for skin grafting, prolonged splinting, and bulky tissue into the defect that minimizes the cosmetic results.^11^,^12^

Local advancement flaps may be considered for elbow soft tissue defect. The use of the local advancement flap is restricted by the required skin grafting.^13^

The microvascular free tissue transfer is rarely indicated for elbow region.^3^

Muscle and musculocutaneous flaps, for example, flexor carpi ulnaris have been described as potentially capable of restoring posterior defects of the elbow.^2^ The selection of these flaps is largely related to muscle volume and rotation arc. If musculocutaneous flaps are used, one should consider the degree of functional loss of the muscle.^2^

The anatomical basis of fasciocutaneous arm flaps have already been described by some researchers, previously.^14^,^15^ All of these articles studied in a wide regions arm perforator flaps, but we were specially focused on distal medial arm region in this study.

The DBAMPFF must be carefully elevated in order to prevent medial brachial cutaneous nerve injury. Chowdhry et al^16^ showed that the medial brachial cutaneous nerve runs with the basilic vein and sends 2 to 4 branches to the skin 7 cm proximal to the medial epicondyle. Another 3 to 5 branches pierce the fascia to innervate the skin at about 15 cm proximal to the medial epicondyle. Knowledge of the nerve course can help the protection of medial brachial cutaneous nerve when the DBAMPFF is elevated.

Local fasciocutaneous flaps such as reverse lateral arm flap^17^ or ulnar recurrent fasciocutaneous flap^6^ may be useful for elbow contracture releasing and soft tissue coverage. Lateral arm flap's main disadvantage is the flap's donor scar to be located on the lateral site of the arm. The scar of the flap (DBAMPFF), we described in this cadaveric study, stays on the distal medial arm. Some authors have described their clinical experiences for reverse medial arm flap raising on the ulnar recurrent artery. We have also experienced reverse medial arm flap to release elbow region defect in many cases, and acceptable results were acquired. But, vascular dissection of the reverse medial arm flap is more difficult than DBAMPFF because its vascular supply comes from the recurrent ulnar artery, which is located in the posterior distal arm.

Recently, perforator flaps have been commonly preferred in various conditions based on the angiosome concept.^18^^-^24 We recognized the distal brachial artery main perforator vessel while raising the ulnar recurrent fasciocutaneous flap. This cadaveric study showed that the distal brachial artery main perforator vessel's diameter might be wide enough, and the vessel's length might be suitable to rotation and local transfer for small-to-moderate size soft tissue defect in elbow. But, the flap's pedicle length might not be enough for large elbow soft tissue reconstruction. We have shown that the DBAMPFF has a consistent main perforator artery at all times, and flap raising is made easily. Practically, several small perforators may be added to DBAMPFF easily when needed, and if they do not limit arc of rotation and transfer.

In conclusion, we believe that the DBAMPFF might be a useful alternative for the elbow soft tissue defects reconstruction. Clinical studies are necessary to highlight the real impact of this flap technique.

## Figures and Tables

**Figure 1:**
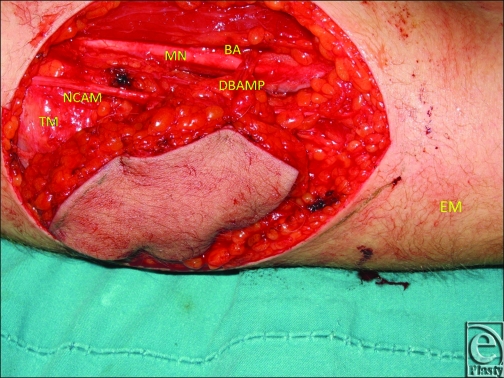
Distal brachial artery's main perforator recognized when ulnar recurrent fasciocutaneous flap was raised. Abbreviations: BA, brachial artery; DBAMP, distal brachial artery's main perforator; EM, epicondylus medialis; MN, median nerve; NCAM, nervus cutaneous antebrachii medialis.

**Table 1:** Characteristics of the distal brachial artery's main perforator fasciocutaneous flap

Cadavers	Right arm (RA)	Left arm (LA)	Perforator arter diameter, mm (RA)	Perforator arter diameter, mm (LA)	Perforator arter length, cm (RA)	Perforator arter length, cm (LA)	Perforator arter distance from medial epicondyle of humerus (RA)	Perforator arter distance from medial epicondyle of humerus (LA)	Skin territory of perforator flap, cm (RA)	Skin territory of perforator flap, cm (LA)
1	X	X	1.17	1.15	3.7	3.4	12.6 cm above and 1.5 cm medial	12 cm above and 1.6 cm medial	11 × 6	10 × 6
2	X	X	0.93	0.87	2.9	3.4	10 cm above and 1.3 cm medial	10 cm above and 1.3 cm medial	10 × 5	11 × 6
3	X	X	0.89	0.86	3	3.1	13 cm above and 1.3 cm medial	12.5 cm above and 1.2 cm medial	11 × 5	10 × 6
4	X	X	0.99	0.92	3.2	3.1	11.8 cm above and 1.4 cm medial	11.5 cm above and 1.3 cm medial	12 × 6	11 × 6
5	X	X	1	0.96	3.6	3.4	12 cm above and 1.2 cm medial	12 cm above And 1.3 cm medial	10.5 × 6	10 × 5
6	X	X	0.87	0.83	3.1	3.6	10.7 cm above and 1.2 cm medial	10 cm above and 1.1 cm medial	11 × 5.5	10 × 6
7	X	X	0.95	0.96	3.3	3.5	11 cm above and 1.2 cm medial	12 cm above and 1.3 cm medial	11 × 6	12 × 5
